# Variability in snake skin microbial assemblages across spatial scales and disease states

**DOI:** 10.1038/s41396-019-0416-x

**Published:** 2019-05-07

**Authors:** Donald M. Walker, Jacob E. Leys, Matthew Grisnik, Alejandro Grajal-Puche, Christopher M. Murray, Matthew C. Allender

**Affiliations:** 1 Toxicology and Disease Group, Biology Department, Middle Tennessee State University, PO Box 60, Murfreesboro, TN USA; 2 Department of Biology, Tennessee Technological University, Cookeville, TN USA; 3 Wildlife Epidemiology Laboratory, College of Veterinary Medicine, University of Illinois at Urbana-Champaign, Champaign, IL USA

**Keywords:** Microbiome, Community ecology

## Abstract

Understanding how biological patterns translate into functional processes across different scales is a central question in ecology. Within a spatial context, extent is used to describe the overall geographic area of a study, whereas grain describes the overall unit of observation. This study aimed to characterize the snake skin microbiota (grain) and to determine host–microbial assemblage–pathogen effects across spatial extents within the Southern United States. The causative agent of snake fungal disease, *Ophidiomyces ophiodiicola*, is a fungal pathogen threatening snake populations. We hypothesized that the skin microbial assemblage of snakes differs from its surrounding environment, by host species, spatial scale, season, and in the presence of *O. ophiodiicola*. We collected snake skin swabs, soil samples, and water samples across six states in the Southern United States (macroscale extent), four Tennessee ecoregions (mesoscale extent), and at multiple sites within each Tennessee ecoregion (microscale extent). These samples were subjected to DNA extraction and quantitative PCR to determine the presence/absence of *O. ophiodiicola*. High-throughput sequencing was also utilized to characterize the microbial communities. We concluded that the snake skin microbial assemblage was partially distinct from environmental microbial communities. Snake host species was strongly predictive of the skin microbiota at macro-, meso-, and microscale spatial extents; however, the effect was variable across geographic space and season. Lastly, the presence of the fungal pathogen *O. ophiodiicola* is predictive of skin microbial assemblages across macro- and meso-spatial extents, and particular bacterial taxa associate with *O. ophiodiicola* pathogen load. Our results highlight the importance of scale regarding wildlife host–pathogen–microbial assemblage interactions.

## Introduction

Understanding biologically meaningful patterns and the scale at which they occur is a central theme in ecology [1, 2]. Incorporating scale (spatial and/or temporal) into an experimental design may allow community ecologists to make predictions or answer applied questions about a system. Macro-ecologists have described distributional structuring of communities [3, 4]; however, host-associated microbial systems seem to lack predictable distributional patterns following the most classical examples [5]. Elucidating the mechanisms that underlie observed patterns across micro- to macroscales, and how they change within the scale of description, was explored here across a microbial system in the southern United States.

Extent and grain are factors used to elucidate ecological spatial scale-based patterns [1]. Scale is a function of both extent, or the overall area encompassed by a study, and grain size is the unit of measurement [1]. Using regional measurements of extent to predict localized effects may confound the interpretation of ecological phenomena. For example, a fine-scale study (small extent) may observe patterns at a fine grain size but produce little understanding of effects across a landscape. On the other hand, studying a broad extent may result in the failure to capture fine grain biological processes, which influence landscape phenomena [1, 6, 7]. In this regard, research incorporating multiple focal scales may help explain how ecological patterns translate into functional processes. Incorporating spatial considerations into hypothesis tests about emerging wildlife pathogens is especially important for understanding how fine to broad landscape patterns may predict host–pathogen–microbial assemblage associations. For example, Allender et al. [8] found that the skin microbiota of the endangered Eastern Massasaugua Rattlesnake (*Sistrurus catenatus*) differed in the presence of the snake fungal disease (SFD) pathogen. However, their study included a single species and encompassed four sites near a single lake in Illinois, USA. Our study took a similar approach to Allender et al. [8] but sampled across a greater host diversity and at different spatial extents.

Numerous species are currently facing global population declines due to a plethora of synergistic factors including anthropogenic-mediated habitat destruction, climate change, and emerging infectious pathogens [9–13]. Although habitat destruction and fragmentation are in part explanatory for species declines, emerging fungal pathogens are of concern for amphibians, reptiles, and bats [10, 12, 13]. Amphibians are suffering declines due to a mycotic infection caused by a chytrid species of fungus *Batrachochytrium dendrobatidis* [14]. This fungus has caused population declines to more than 520 species of frogs, toads, salamanders, newts, and caecilians [15] in biodiversity hotspots globally (e.g., Central America and Australia) [16–19]. Snakes have been affected by the fungal pathogen *Ophidiomyces ophiodiicola*, which has been linked to a syndrome diagnosed as SFD [20, 21]. Population declines due to *O. ophiodiicola* have yet to be clearly documented. However, *O. ophiodiicola* has been suggested as present in a declining population of Timber Rattlesnakes (*Crotalus horridus* [22]) and has been confirmed in a threatened population of Eastern Massasauga Rattlesnakes (*S. catenatus* [23]).

Although the fungus *O. ophiodiicola* has recently been determined as the causative agent of SFD [21, 24], there is limited information regarding the characteristics and life history of this pathogen. Recently, a series of in vitro experiments demonstrated the wide range of environmental parameters for *O. ophiodiicola* and the ability of this fungus to utilize complex carbon, nitrogen, and sulfur resources [25]. The authors hypothesized that *O. ophiodiicola* primarily infects snakes and can persist in the soil, although researchers have yet to find evidence in a natural system.

The microbiome functions as a part of the innate immune system in vertebrates [26–28]. The host microbiome may confer disease resistance from pathogenic fungi by producing antifungal metabolites, outcompeting the fungus for space, or by stabilizing the microbial community to drive defense efficacy [27]. The skin microbial assemblage of snakes remains relatively unstudied and little is known regarding the composition and diversity of bacteria that compose the snake skin microbial assemblage; thus, its characterization is critical to better understanding host–pathogen–microbial assemblage interactions across space and time.

We hypothesized that the snake skin microbial assemblage differed from environmental microbial assemblages, by host species, geographic region, season, and across micro- to macroscales. We postulated that the microbiota of snakes correlates with the presence of *O. ophiodiicola*, and that particular bacteria may correlate with higher pathogen loads. Our objectives were to: (1) determine whether environmental microbes, host species, geographic region, and/or season was predictive of snake microbial assemblages; (2) assess fine to broad scale spatial patterns of snake skin microbial assemblages; (3) determine whether the presence of a fungal pathogen is predictive of snake skin microbial assemblages; and (4) correlate particular bacterial taxa with *O. ophiodiicola* pathogen load.

## Methods

### Field collections

**Fig. 1:**
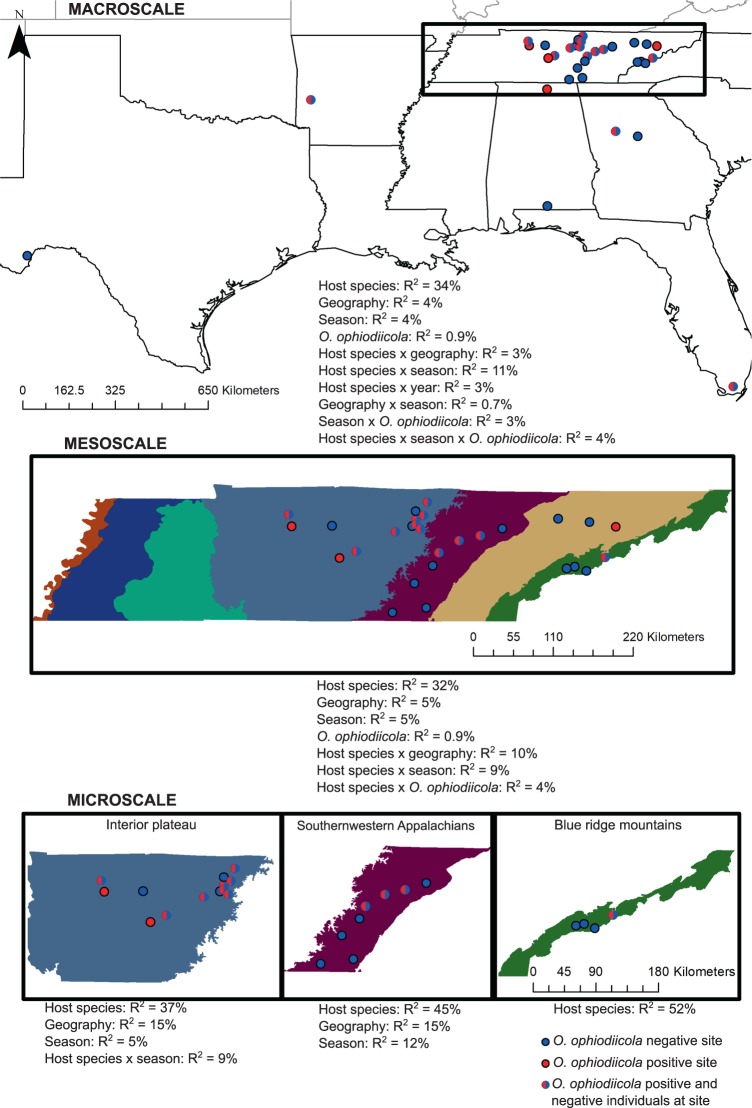
Host species, geography, season, and pathogen presence are predictive of the microbiome across macro- to microspatial scales. Snake sampling sites and presence of *O*. *ophiodiicola* are marked with colored circles across the map. Statistically significant explanatory variables from *adonis* models are listed with the corresponding coefficient of determination (*R*^2^)

During the field seasons of 2015–2017, samples were collected from 35 sites across six states in the Southern United States: Alabama, Arkansas, Florida, Georgia, Tennessee, and Texas (Fig. 1). We defined “macroscale” patterns of microbial assemblages as those observed in the Southern United States. Sampling emphasis focused on four Tennessee ecoregions (“mesoscale”), incorporating 28 sites across the state (“microscale”), to provide a finer scale understanding of the snake skin microbial assemblage (Fig. 1). All snake individuals encountered were captured and handled with clean nitrile gloves and transient microbes were removed by rinsing with 100 mL of sterile deionized water [28, 29] autoclaved for 2 h [30]. A single skin swab was collected using a sterile rayon-tipped swab (Puritan, VWR cat #10808-146) for later DNA extraction. Each snake was swabbed in a way to standardize “grain size” by taking 15 swab strokes along a 15 cm length of the middle-third body section of the animal, encompassing the ventral, dorsal, and lateral body sites. No snakes were collected or sampled that failed to exceed swabbing length.

### Environmental sampling

To assay the environment for *O*. *ophiodiicola*, soil samples (*n* = 48) were procured from the exact point of snake capture, excluding high traffic roads/paths. Soil samples were obtained by aseptically swabbing the soil in concentric circles not exceeding 30 cm in diameter with a rayon-tipped sterile applicator. Samples were immediately stored in dry 2 mL microcentrifuge tubes. To assay the environment for *O*. *ophiodiicola*, water samples (*n* = 29) were collected at the nearest accessible water source (e.g., stream bank adjacent to point capture) in proximity to captured aquatic snakes by aseptically filtering 500 mL of water through a Thermo Scientific Nalgene Analytical Test Funnel (CN 145-2020) with a measured pore size of 0.2 μm. Water was drawn through the filter by a peristaltic pump and the filter paper was aseptically placed into a dry 2 mL centrifuge tube. All three sample types (skin, soil, and water) were immediately stored at −10 °C in a CoolFreeze CF-25 vehicle freezer and transferred directly to a lab freezer at −20 °C until further processing.

### DNA extraction and quantitative PCR

DNA was extracted from filter paper (1/4 slice), skin, and soil swab samples using the Qiagen DNeasy PowerSoil HTP 96 kit, per the manufacturer’s standard protocol. A single DNA extraction negative control blank was extracted on each plate of 96 samples or as a single preparation when samples exceeded the 96-well plate. All negative controls were screened using quantitative PCR (qPCR) and/or high-throughput DNA sequencing. To minimize cross-contamination, DNA extraction, qPCR, and library setup were done in separate AirClean Systems AC600 (AirClean Systems, Creedmoor, NC) dedicated to these processes. Each workstation had a dedicated set of pipettes that were routinely autoclaved before experimentation. DNA was concentrated to ≈20 µl using a Thermo Fisher Savant DNA SpeedVac. The molecular presence of *O*. *ophiodiicola* in all skin and environmental samples was detected using qPCR. The qPCR assay as described by Bohuski et al. [31] was used to amplify the internal transcribed spacer region of the rRNA gene of *O*. *ophiodiicola*. Each 96-well qPCR sample plate was processed with a positive control and two no-template negative control reactions. Sample reactions were run in triplicate and consisted of a 10 μL volume containing 5 μL IDT 2× Primetime MasterMix, 0.4 μL of IDT forward primer (10 μM), 0.4 μL of IDT reverse primer (10 μM), 0.1 μL (20 μM) IDT probe, 2.1 μL PCR-grade water, and 2 μL of DNA template. Thermocycling conditions consisted of a 3 min cycle at 95 °C, followed by 50 cycles of 95 °C for 10 s and 60 °C for 30 s. A positive test was confirmed by an exponential phase appearing at a *C*_t_ < 39 in each reaction. Samples showing incomplete evidence by testing positive for one or two replicates during the first run were reanalyzed on a separate plate. Samples still showing ambiguous results (one or two positive reactions) after the second round of amplification were considered positive [32–34], whereas samples not amplifying in triplicate on the second plate were considered negative. To calculate pathogen load from resulting *C*_t_ values, a single standard curve was run using a serial dilution of 1 × 10^10^ − 1 amplicon copies of a synthetic gBlocks (Integrated DNA Technologies) fragment identical to the target qPCR region in Bohuski et al. [31]. The standard curve was used to generate the formula, *y* = −0.2915 × +11.094, where “*x*” is average *C*_t_ value of each sample, and this was used to calculate the log copy number (herein, “pathogen load”) of the ITS region of the rRNA gene of *O*. *ophiodiicola* per qPCR reaction. All *C*_t_ values and resulting pathogen load values are reported in Supplementary Data File 1.

### High-throughput sequencing and bioinformatics analyses

A subset of 168 skin swabs, ten soil samples from TN, and ten water samples from TN were selected for high-throughput sequencing. These samples represented a cross-sectional view of samples collected across 35 field sites and all three years. Sequencing was performed according to the Illumina 16S Metagenomic Sequencing Library Preparation protocol on the Illumina MiSeq instrument in two separate sequencing runs. The V4 region of the 16S rDNA was amplified using dual indexed fusion primers as described by Kozich et al. [35]. Samples were cleaned using Ampure XP magnetic beads after both the initial PCR and index PCR step. After amplification and indexing, PCR products were quantified on a Qubit fluorometer 3.0, per the manufacturer’s protocol, and visualized for amplicon size (≈450 bp) on an Agilent 2100 Bioanalyzer according to the DNA 1000 protocol, and then normalized. After library quality control and quantification, the library was loaded on an Illumina MiSeq v3 flow cell and sequenced using a 500-cycle reagent kit (paired-end 2 × 300 reads). A total of 13,908,315 raw data sequence reads were obtained during run one and 10,137,882 in run two. Data were processed per the MiSeq SOP described by Kozich et al. [35] using mothur v1.39.5. After assembling paired-end reads into contigs, sequences were removed from the analysis if they had fewer than 249 bp or >253 bp, contained homopolymers in excess of eight nucleotides, or contained any ambiguous base calls. Of these, unique sequences were aligned to the SILVA v.123 bacterial reference database [36] curated to the V4 region of interest, pre-clustered allowing for two nucleotide differences, and chimeras removed per the mothur UCHIME algorithm [37]. Remaining alignments were classified into taxonomic lineage using *classify.seqs* at an 80% cutoff value, and lineages identified as Archaea, Eukaryota, chloroplast, mitochondria, and unknown were removed [35]. Sequences passing all quality control metrics were clustered into operational taxonomic units (OTUs) using *cluster.split* at 97% sequence similarity [38]. Rare OTUs appearing five times or fewer throughout the dataset were removed [39]. In addition, 64 OTUs appearing in the negative control DNA extraction blanks (1,376 reads) were removed from the dataset. A total of 8,654,686 sequence reads were kept after filtering and utilized in all downstream analyses. Ten skin swab samples were removed (*n* = 178 total samples retained) due to inadequate coverage after data filtering and curation in mothur. To normalize, we ran *summary.single* in mothur to compute sample coverage, then subsampled at 2,101 sequence reads per sample, and used this dataset in statistical analyses. Code to reproduce the bioinformatics analysis is provided in the Supplementary “mothur code” File.

### Statistical analyses—comparison of the skin with environment

All analyses and graphing were conducted in mothur v1.39.5 and R version 3.5.1 using the packages *vegan* [40], *plyr* [41], *dplyr* [42], *ggplot2* [43], *rcompanion* [44], *car* [45], and *gridExtra* [46]. To test whether the snake skin microbial assemblages differed from the environment, the skin, soil, and water bacterial OTU beta diversity was calculated using the *vegdist* function to generate a Bray–Curtis dissimilarity matrix representing sample-to-sample pairwise distances, and these distances were further analyzed using the *metaMDS* function to generate a non-metric multidimensional scaling (nMDS) ordination. The *adonis* function was used to perform a permutational multivariate analysis of variance using 999 permutations on the Bray–Curtis dissimilarity matrix, to determine whether skin, soil, or water were explanatory variables for OTU assemblages.

### Comparisons of the skin microbial assemblages across spatial extents

As the skin assemblage of snakes differed from the environmental microbial assemblages (see Results below), we used an indicator analysis (indicator values > 30, *p* *<* 0.05) to select a subset of 56 OTUs that were descriptive of the variation in the snake skin microbial assemblages across all collected skin samples. To understand the effect of spatial scale on the snake skin microbial assemblages, we subdivided our dataset into three categories for analysis including the following: (1) broad-scale patterns (macroscale) in the Southern United States (*n* = 158 samples); (2) mesoscale patterns across four geographically distinct Tennessee ecoregions (*n* = 124 samples); (3) microscale patterns of between site variation within each Tennessee ecoregion (Fig. 1). For the macroscale dataset, all samples were coded as occurring in a geographic region (Alabama, Arkansas, Florida, Georgia, Tennessee, or Texas). Tennessee ecoregion (mesoscale) factors included the blue ridge mountains, interior plateau, ridge and valley, and Southwestern Appalachians. Microscale categories included four sites within the blue ridge mountains (*n* = 16 skin samples), 13 sites within the interior plateau (*n* = 73 skin samples), and 8 sites within the Southwestern Appalachians (*n* = 32 skin samples). Site-based differences within the ridge and valley ecoregion were not analyzed due to a small sample size effect (three sites, three samples). The *adonis* function was used to perform a permutational multivariate analysis of variance using 999 permutations on the Bray–Curtis dissimilarity matrix of 56 indicator OTUs to test whether the explanatory variables including host species, geographic location, season, year, *O*. *ophiodiicola* status (positive/negative), or interactions between these factors were predictive of microbiota diversity at micro-, meso-, and macroscales. We also evaluated alpha diversity as a tentative variable component of snake skin microbial assemblages by comparing inverse Simpson diversity values for the explanatory variables host species and *O*. *ophiodiicola* status (positive/negative) using a Kruskal–Wallis test across micro-, meso-, and macroscales.

### Host life history and the skin microbiota

“Ecomode” is defined as modal categories (e.g., arboreal, aquatic, fossorial, and terrestrial) of habitat use in species that do not necessarily display convergent morphology [47]. To determine whether snake life history was predictive of microbial assemblages, all snake species were categorized by ecomode including aquatic, arboreal, fossorial, or terrestrial, and an nMDS ordination was run using the *metaMDS* function on Bray–Curtis dissimilarity matrix values for each micro- to macroscale dataset of the 56 previously described indicator OTUs. The *adonis* function in vegan was used to perform a permutational multivariate analysis of variance using 999 permutations on the Bray–Curtis dissimilarity matrix of indicator OTUs to test whether the explanatory variables including ecomode, *O*. *ophiodiicola* status, or an interaction between these factors was predictive of microbiome diversity at micro-, meso-, and macroscales. We assessed the alpha diversity of ecomode categories using a Kruskal–Wallis test on inverse Simpson diversity values across micro-, meso-, and macroscales.

### Pathogen load and the skin microbiota

To understand the structure of the microbiota in the presence of a fungal pathogen across space, we performed indicator analyses (indicator values > 30, *p* *<* 0.05) in mothur to select OTUs descriptive of the skin microbial assemblage in the presence/absence of *O*. *ophiodiicola* independently across macro-, meso-, and microscales. We conducted a constrained analysis of proximities using the *capscale* function in vegan to model the continuous explanatory variable, *O*. *ophiodiicola* pathogen load (qPCR results), on Bray–Curtis dissimilarity values from microbial assemblages of *O*. *ophiodiicola*-positive/negative snakes. Both *O*. *ophiodiicola* pathogen load/reaction and OTU relative abundances were cube root transformed before the *capscale* analysis. We modeled the correlation between *O*. *ophiodiicola* pathogen load and microbial assemblages for 16 indicator OTUs at the macroscale, 17 OTUs at the mesoscale, 12 OTUs for the interior plateau (microscale), and 12 OTUs for the Southwestern Appalachian mountain ecoregion (microscale). We did not model the described associations for the ridge and valley ecoregion, as we only collected one *O*. *ophiodiicola* positive snake from this location. The complete list of samples processed, taxonomic identifications, and statistical models is presented in Supplementary Tables 1–3. See Grisnik et al. [48] for information about clinical signs for a subset of snakes used in these analyses.

## Results

### Distribution of *O. ophiodiicola*

We detected the presence of *O*. *ophiodiicola* DNA on 43 snake individuals from 13 species and at 19 field sites. Of all soil samples collected (*n* = 48), two locations on the landscape tested positive for DNA of *O*. *ophiodiicola* at Waterloo Falls, TN (*C*_t_ = 36.3, copies/rxn = 3.2) and Edgar Evins State Park, TN (*C*_t_ = 37.3, copies/rxn = 1.6) [Supplementary Data File 1]. Positive soil samples correlated with point capture locations for *O*. *ophiodiicola*-positive animals. Of all water samples (*n* = 29), zero tested positive for *O*. *ophiodiicola* DNA.

### Comparison of snake skin microbial assemblages with the environment

**Fig. 2:**
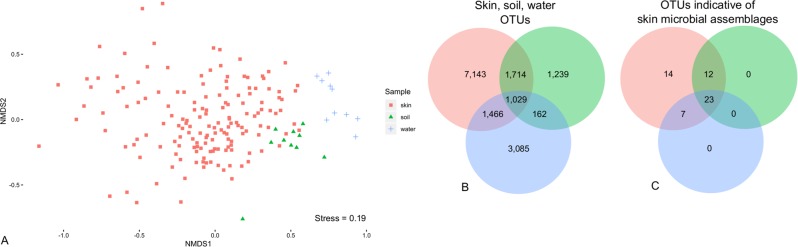
**a** Beta diversity patterns of the snake skin microbial assemblage compared with environmental microbiota from both soil and water, visualized using a non-metric multidimensional scaling ordination. **b** Venn diagram showing overlap of OTUs between skin (red), soil (green), and water (blue) samples. **c** Venn diagram showing results from indicator analysis selecting for OTUs that describe variation in the snake skin microbial assemblage

Analysis of the three sample types indicated that the skin microbial assemblage of individual snakes hosted between 25 and 824 OTUs (mean = 250, SD = 187, *n* = 158), whereas individual soil samples (range = 382–991 OTUs, mean = 786, SD = 173, *n* = 10) and water samples (range = 582–1,294 OTUs, mean = 966, SD = 230, *n* = 10) hosted more species-rich communities. To determine how well we sampled each community based on the rarefied data, we calculated sequence sample coverage for all samples (55% outlier, range = 61–99%, mean = 89%, SD = 8.7%). We determined that the snake skin microbial assemblage was distinct from the surrounding environment (*F*_2, 177_ = 4.01, *p* *<* 0.001, *R*^2^ = 5.4%; Fig. 2a, Supplementary Table 1). Although the beta diversity of skin, soil, and water differed, the three community types shared 1,029 OTUs (Fig. 2b). Furthermore, 23 of the 56 OTUs selected by the indicator analysis as describing significant variation in the snake skin microbial assemblage are shared with the environment (Fig. 2c).

### Comparisons of the skin microbial assemblages across spatial extents

At the macroscale (Southern United States), host snake species (*F*_20, 157_ = 4.22, *p* *<* 0.005, *R*^2^ = 33.7%), geography (*F*_4, 157_ = 2.27, *p* *<* 0.005, *R*^2^ = 3.6%), season (*F*_5, 157_ = 2.19, *p* *<* 0.005, *R*^2^ = 4.4), and *O*. *ophiodiicola* status (*F*_1, 157_ = 2.33, *p* *<* 0.005, *R*^2^ = 0.9%) were predictive of the snake skin microbial assemblage (Fig. 1, Supplementary Table 1). The effect of season on the skin microbiota differed by snake species (*F*_19, 157_ = 1.48, *p* *<* 0.005, *R*^2^ = 11.3%) and this varied further by *O*. *ophiodiicola* status (*F*_9, 157_ = 1.23, *p* *<* 0.05, *R*^2^ = 4.4%). Season also had a differential effect on the skin microbial assemblage across geographic space (*F*_1, 157_ = 1.78, *p* *<* 0.05, *R*^2^ = 0.7%). Both geographic location (*F*_5, 157_ = 1.29, *p* *<* 0.05, *R*^2^ = 2.6%) and sampling year (*F*_7, 157_ = 1.23, *p* *<* 0.05, *R*^2^ = 3.4%) had a varied effect on independent host species’ microbial assemblages. At the macroscale, alpha diversity differed by host species (*χ*^2^ [20] = 61.4, *p* *<* 0.001) but not *O*. *ophiodiicola* status (*χ*^2^ [1] = 1.26, *p* > 0.05) [Supplementary Table 1].

At the mesoscale (Tennessee ecoregion), host snake species (*F*_16, 123_ = 3.78, *p* *<* 0.005, *R*^2^ = 31.9%), geography “ecoregion” (*F*_3, 123_ = 3.23, *p* < 0.005, *R*^2^ = 5.1%), season (*F*_5, 123_ = 1.82, *p* < 0.005, *R*^2^ = 4.8%), and *O*. *ophiodiicola* status (*F*_1, 123_ = 1.90, *p* < 0.05, *R*^2^ = 0.9%) were predictive of the skin microbial assemblages. We determined that geography “ecoregion” (*F*_13, 123_ = 1.44, *p* < 0.005, *R*^2^ = 9.8%), season (*F*_14, 123_ = 1.26, *p* < 0.005, *R*^2^ = 9.3%), and *O*. *ophiodiicola* status (*F*_7, 123_ = 1.22, *p* < 0.05, *R*^2^ = 4.4%) all have a differential effect on independent host snake species microbial assemblages [Supplementary Table 1]. At the mesoscale, alpha diversity varied by host species (*χ*^2^ [16] = 44.4, *p* *<* 0.001) but not *O*. *ophiodiicola* status (*χ*^2^ [1] = 0.67, *p* > 0.05) [Supplementary Table 1].

At the microscale, host snake species was predictive of the skin assemblage for the interior plateau (*F*_14, 72_ = 2.93, *p* *<* 0.005, *R*^2^ = 37.4%), Southwestern Appalachians (*F*_7, 31_ = 3.60, *p* *<* 0.005, *R*^2^ = 45.4%), and blue ridge mountains (*F*_6, 15_ = 1.44, *p* *<* 0.05, *R*^2^ = 51.8%). Both geography “site” and season were predictive of the skin microbial assemblage for the interior plateau (geography: *F*_11, 72_ = 1.48, *p* *<* 0.005, *R*^2^ = 14.8%, season: *F*_4, 72_ = 1.49, *p* *<* 0.01, *R*^2^ = 5.4%) and Southwestern Appalachians (geography: *F*_6, 31_ = 1.37, *p* *<* 0.05, *R*^2^ = 14.8%, season: *F*_3, 31_ = 2.24, *p* *<* 0.005, *R*^2^ = 12.1%) but not the blue ridge mountains (geography: *F*_1, 15_ = 0.98, *p* > 0.05, season: *F*_1, 15_ = 0.70, *p* > 0.05). The effect of season on the skin microbial assemblage differed by host species (*F*_7, 72_ = 1.47, *p* *<* 0.005, *R*^2^ = 9.4) within the interior plateau. There were no statistically significant interactions between explanatory variables for the Southwestern Appalachians or blue ridge mountain ecoregions (Supplementary Table 1). The presence of *O*. *ophiodiicola* was not predictive of skin microbial assemblages for the interior plateau (*F*_1, 72_ = 1.19, *p* > 0.05) or Southwestern Appalachians (*F*_1, 31_ = 1.22, *p* > 0.05) and was not tested in the blue ridge mountains due to small sample sizes. Alpha diversity differed by host species within the interior plateau (*χ*^2^ [14] = 26.4, *p* *<* 0.05) and Southwestern Appalachian ecoregions (*χ*^2^ [7] = 14.8, *p* *<* 0.05) but not the blue ridge mountains (*χ*^2^ [6] = 10.6, *p* > 0.05). Alpha diversity did not differ by *O*. *ophiodiicola* status within the interior plateau (*χ*^2^ [1] = 0.18, *p* > 0.05) or Southwestern Appalachian ecoregions (*χ*^2^ [1] = 3.2, *p* > 0.05).

### Host life history and the skin microbiota

**Fig. 3:**
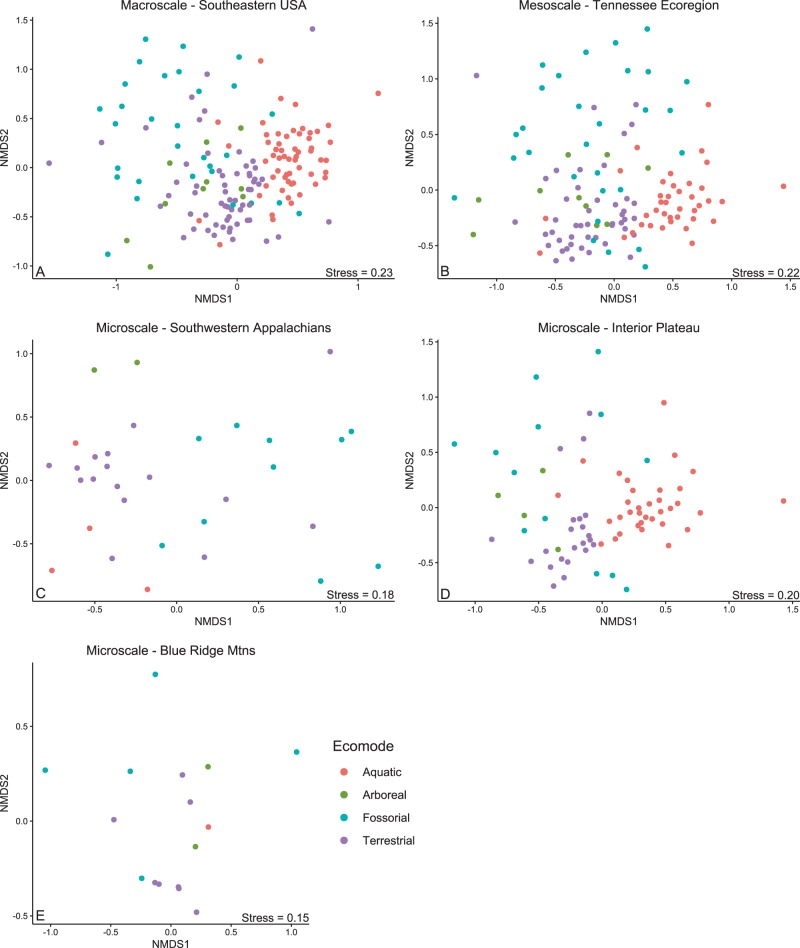
Beta diversity patterns of the snake skin microbial assemblage compared by ecomode and visualized using a non-metric multidimensional scaling ordination. **a** Macroscale—Southern United States. **b** Mesoscale—Tennessee ecoregions. **c** Microscale—Southwestern Appalachians ecoregion. **d** Microscale—Interior plateau ecoregion. **e** Microscale—Blue ridge mountains ecoregion

Snake ecomode was strongly predictive of skin microbial assemblages at the macro-, meso-, and microscales (interior plateau; Fig. 3). At the macroscale, ecomode (*F*_3, 157_ = 8.10, *p* *<* 0.005, *R*^2^ = 13.3%) was predictive of microbial assemblages and *O*. *ophiodiicola* presence had a variable effect within independent ecomodes (*F*_3, 157_ = 1.73, *p* *<* 0.005, *R*^2^ = 2.8%)[Supplementary Table 1]. Similarly, at the mesoscale (Tennessee ecoregions), ecomode (*F*_3, 123_ = 6.32, *p* *<* 0.005, *R*^2^ = 13.3%) was predictive of skin microbial assemblages and this effect differed by *O*. *ophiodiicola* status within independent ecomodes (*F*_3, 123_ = 1.59, *p* *<* 0.005, *R*^2^ = 3.3%). Likewise, at the microscale (interior plateau), ecomode (*F*_3, 72_ = 4.56, *p* *<* 0.005, *R*^2^ = 15.9%) was predictive of the skin microbial assemblage and *O*. *ophiodiicola* status had a differing effect within independent ecomodes (*F*_3, 72_ = 1.57, *p* *<* 0.05, *R*^2^ = 5.5%). Ecomode was also predictive of the skin microbial assemblage at the microscale within the Southwestern Appalachians (*F*_3, 31_ = 2.63, *p* *<* 0.005, *R*^2^ = 21.8%) but not in the blue ridge mountain ecoregion (*F*_3, 15_ = 1.27, *p* > 0.05).

### Fungal pathogen load and the skin microbiota

**Fig. 4:**
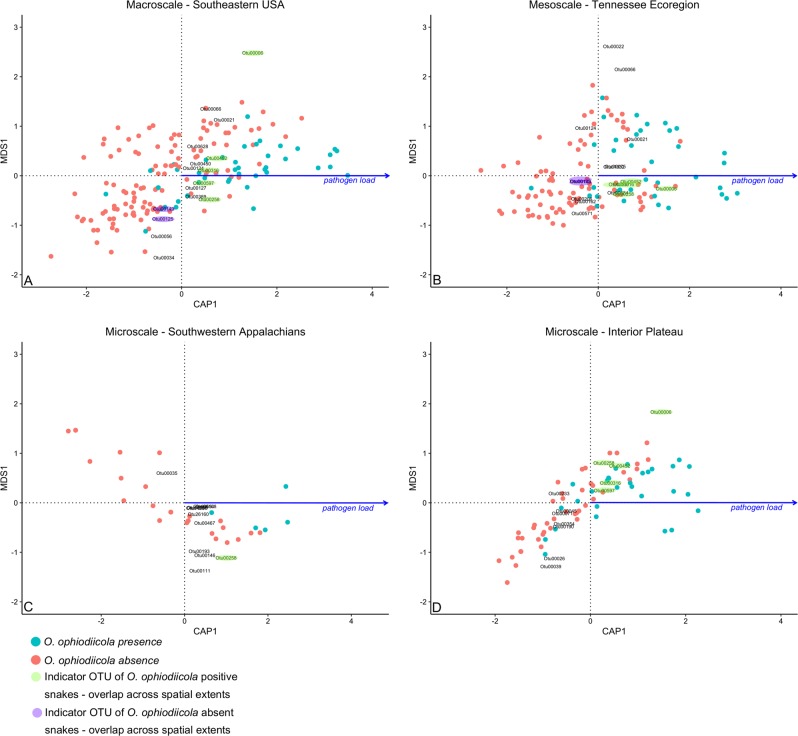
Results depicting constrained analysis of proximities to model the continuous explanatory variable, *O*. *ophiodiicola* pathogen load (qPCR results), on Bray–Curtis dissimilarity values from skin microbial assemblages of *O*. *ophiodiicola*-positive/negative snakes. Taxonomic affiliation of OTU (e.g., Otu00006) labels is found in Table 1. Colored circles show the microbial assemblage of *O*. *ophiodiicola*-positive/negative individuals. Colored ovals show OTUs that correlate with pathogen load across macro-, meso-, and microscale spatial extents. The blue vector labeled “pathogen load” shows the direction of the relationship between higher fungal load, snake skin microbial assemblages, and indicator OTUs

The amount of the SFD pathogen, determined by number of ITS copies per reaction (pathogen load), was correlated with specific microbial taxa (Table 1). Five OTUs are explanatory of higher *O*. *ophiodiicola* pathogen load across macro-, meso-, and microscales (Fig. 4a, b, d). Similarly, one OTU (Otu00258) was predictive of higher *O*. *ophiodiicola* pathogen load across all four spatial scales tested (Fig. 4a–d). Two OTUs (Otu00125 and Otu00149) were correlated with *O*. *ophiodiicola* absence across both macro- and mesoscales (Fig. 4a, b).

**Table 1:** Taxonomy of OTUs indicative of *O*. *ophiodiicola* status and fungal load

OTU	Phylum	Class	Order	Family	Genus
Otu00233	Actinobacteria	Actinobacteria	Acidimicrobiales	Unclassified	Unclassified
**Otu00258**	Actinobacteria	Actinobacteria	Actinomycetales	Micrococcaceae	Arthrobacter
Otu00571	Actinobacteria	Actinobacteria	Actinomycetales	Nakamurellaceae	Unclassified
Otu00450	Actinobacteria	Actinobacteria	Actinomycetales	Nocardiaceae	Rhodococcus
Otu00389	Actinobacteria	Actinobacteria	Actinomycetales	Nocardiaceae	Williamsia
*Otu00125*	Actinobacteria	Actinobacteria	Actinomycetales	Nocardioidaceae	Nocardioides
Otu00034	Actinobacteria	Actinobacteria	Actinomycetales	Unclassified	Unclassified
*Otu00149*	Actinobacteria	Actinobacteria	Actinomycetales	Unclassified	Unclassified
Otu00026	Bacteroidetes	Flavobacteria	Flavobacteriales	Flavobacteriaceae	Flavobacterium
Otu00124	Bacteroidetes	Sphingobacteria	Sphingobacteriales	Cytophagaceae	Hymenobacter
Otu00193	Bacteroidetes	Sphingobacteria	Sphingobacteriales	Cytophagaceae	Hymenobacter
Otu10700	Bacteroidetes	Sphingobacteria	Sphingobacteriales	Cytophagaceae	Spirosoma
Otu00206	Bacteroidetes	Sphingobacteria	Sphingobacteriales	Sphingobacteriaceae	Mucilaginibacter
**Otu00316**	Bacteroidetes	Sphingobacteria	Sphingobacteriales	Sphingobacteriaceae	Pedobacter
**Otu00452**	Bacteroidetes	Sphingobacteria	Sphingobacteriales	Sphingobacteriaceae	Sphingobacterium
Otu00022	Deinococcus-Thermus	Deinococci	Deinococcales	Deinococcaceae	Deinococcus
Otu00354	Deinococcus-Thermus	Deinococci	Deinococcales	Deinococcaceae	Deinococcus
Otu00075	Proteobacteria	Alphaproteobacteria	Caulobacterales	Caulobacteraceae	Brevundimonas
**Otu00597**	Proteobacteria	Alphaproteobacteria	Rhizobiales	Hyphomicrobiaceae	Devosia
Otu00111	Proteobacteria	Alphaproteobacteria	Rhizobiales	Methylobacteriaceae	Methylobacterium
Otu00162	Proteobacteria	Alphaproteobacteria	Rhizobiales	Rhizobiaceae	Rhizobium
Otu00045	Proteobacteria	Alphaproteobacteria	Rhizobiales	Unclassified	Unclassified
Otu00190	Proteobacteria	Alphaproteobacteria	Rhizobiales	Unclassified	Unclassified
**Otu00006**	Proteobacteria	Alphaproteobacteria	Rhodobacterales	Rhodobacteraceae	Paracoccus
Otu01032	Proteobacteria	Alphaproteobacteria	Rhodobacterales	Rhodobacteraceae	Paracoccus
Otu00039	Proteobacteria	Alphaproteobacteria	Rhodobacterales	Rhodobacteraceae	Unclassified
Otu00066	Proteobacteria	Alphaproteobacteria	Rhodobacterales	Rhodobacteraceae	Unclassified
Otu00467	Proteobacteria	Alphaproteobacteria	Rhodospirillales	Acetobacteraceae	Roseomonas
Otu00056	Proteobacteria	Alphaproteobacteria	Sphingomonadales	Sphingomonadaceae	Sphingomonas
Otu00146	Proteobacteria	Alphaproteobacteria	Sphingomonadales	Sphingomonadaceae	Sphingomonas
Otu00035	Proteobacteria	Alphaproteobacteria	Sphingomonadales	Sphingomonadaceae	Sphingosinicella
Otu00684	Proteobacteria	Betaproteobacteria	Burkholderiales	Alcaligenaceae	Unclassified
Otu00108	Proteobacteria	Betaproteobacteria	Burkholderiales	Comamonadaceae	Hydrogenophaga
Otu01591	Proteobacteria	Betaproteobacteria	Burkholderiales	Comamonadaceae	Unclassified
Otu00071	Proteobacteria	Gammaproteobacteria	unclassified	Unclassified	Unclassified
Otu00127	Proteobacteria	Gammaproteobacteria	Xanthomonadales	Xanthomonadaceae	Unclassified
Otu00628	Proteobacteria	Gammaproteobacteria	Xanthomonadales	Xanthomonadaceae	Unclassified
Otu00554	Unclassified	Unclassified	Unclassified	Unclassified	Unclassified
Otu14287	Unclassified	Unclassified	Unclassified	Unclassified	Unclassified
Otu26160	Unclassified	Unclassified	Unclassified	Unclassified	Unclassified

Taxa with italicized text correlate with the absence of *O*. *ophiodiicola* across both macro- to mesoscales. Taxa in bold text correlate with *O*. *ophiodiicola* presence and fungal load across macro-, meso-, and microscales. All other OTUs were determined as indicator taxa of *O*. *ophiodiicola* but did not show a pattern across spatial scales

## Discussion

These results help to elucidate the distribution of *O*. *ophiodiicola* across the Southern United States, in particular with fine-scale resolution across Tennessee. Further, they illustrate that *O*. *ophiodiicola* DNA is found in the soil, the skin bacterial assemblage is unique to the host snake species, partially distinct from the environment, and variable between host niche. Specific to our objectives, these data demonstrate that (1) snake species was a significant and strong predictor of the skin microbial assemblage across regions of the Southern United States (macroscale), within-state ecoregions (mesoscale), and within independent ecoregions (microscale); (2) the fungal pathogen *O*. *ophiodiicola* was also a strong predictor of snake skin microbial assemblages for certain hosts across macro- and mesoscales but not at the microscale; (3) snake ecomode was indicative of the skin microbial assemblages across spatial scales with *O*. *ophiodiicola* having a differential effect on microbial assemblages by ecomode; and (4) pathogen load of *O*. *ophiodiicola* correlated with certain bacterial OTUs across spatial scales.

Host snake species was a significant predictor of the skin microbial assemblage across all scales, although the effect varied across both geographic location and season. From this result, we inferred that snake species are associated with taxon-specific skin bacterial assemblages and snake behavior may cause microbial shifts on a temporal (seasonal) basis across the landscape. Similar skin microbial assemblage patterns have been observed in both tropical and temperate amphibian species [49, 50] with similarities pertaining to antifungal metabolites that may protect the host against emerging fungal pathogens, such as *Batrachochytrium salamandrivorans* or *B. dendrobatidis* [28, 51–57]. Similar to our study, in snakes, Hill et al. [58] identified unique bacterial and fungal assemblages from the skin of North American Racers (*Coluber constrictor*) that differed from Timber Rattlesnakes (*C. horridus*). Snake microhabitat preference, seasonal physiological shifts, or environmental differences between spatial extents likely all contribute to the strong effect observed between host species and the skin microbial assemblages.

The fungal pathogen *O*. *ophiodiicola* was also strongly predictive of the host snake species skin microbial assemblages, although this effect was species specific and seasonal. Interestingly, the presence of *O*. *ophiodiicola* correlated with beta- but not alpha-diversity of the skin microbial assemblages across spatial scales. Perhaps this can be explained by the addition (via infection) of *O*. *ophiodiicola* to the skin creating a less stable environment, shifting beta- (turnover and colonization), but not alpha-diversity of microbial assemblages.  This result is of conservation concern, as the host skin microbial assemblage may influence susceptibility to wildlife pathogens such as *O*. *ophiodiicola*. The presence of *O*. *ophiodiicola* coincides with changes in the abundance and composition of the native microbial assemblages found on snake skin. Similarly, Lemieux-Labonté et al. [59] and Allender et al. [8] found that both bacterial and fungal assemblages correlate with the presence of a pathogen in the bat and snake skin microbial assemblage, respectively. This illustrates an interesting parallel of disturbance, whether physical or pathogen mediated, which correlates with alterations in the skin microbial assemblage of a host. Moreover, this is one of the few studies to show that the presence of a wildlife pathogen on the skin of snakes may influence the host’s skin microbial assemblage. The question remains about whether *O*. *ophiodiicola* drives changes in skin microbial assemblages or a disturbance occurs, and the pathogen capitalizes. Alternatively, perhaps the skin microbiota respond to pathogenicity via a change in community composition. Equally likely are seasonal changes in behavior, immunological response, and thermoregulatory requirements being responsible for the differences in the skin microbial assemblages, and no direct relation to disease at all. Future lab-based manipulation of the snake skin microbial assemblages in the presence of *O*. *ophiodiicola* will permit tests of these hypotheses.

Particular bacterial members of the snake skin microbiota correlated with fungal pathogen load across spatial scales. Two OTUs in the Actinobacteria, including *Nocardioides* sp. and an unknown taxon were predictive of *O*. *ophiodiicola* absence across macro- to meso- spatial scales. Interestingly, a species of *Nocardioides* was documented with antifungal activity on salamander skin [28]. From this, we may hypothesize that *Nocardioides* species are important members of the snake skin microbial assemblage with potential for antifungal functional activity across the spatial landscape. Two OTUs in the Bacteroidetes (*Pedobacter* sp. and *Sphingobacterium* sp.), two in the Proteobacteria (*Devosia* sp. and *Paracoccus* sp.), and one in the Actinobacteria (*Arthrobacter* sp.) correlated with higher *O*. *ophiodiicola* fungal loads across macro-, meso-, and micro-spatial scales. The Proteobacteria have been documented as a core members of the skin microbiota of a variety of Panamanian frogs [57], fishes [60], and Appalachian salamanders [61]. Proteobacteria account for a great range of diversity and function, including pathogens and free-living organisms [62]. Similar to our study, Allender et al. [8] found that Eastern Massasauga Rattlesnakes with SFD had an increased relative abundance of *Paracoccus* sp. and *Sphingobacterium* sp. The OTUs that we identified as *Paracoccus* sp. (Otu00006) and *Sphingobacterium* sp. (Otu00452) showed the strongest correlative pattern with high pathogen loads in *O*. *ophiodiicola*-positive individuals. Perhaps, most interestingly, the indicator OTUs (*Pedobacter* sp., *Sphingobacterium* sp., *Devosia* sp., *Paracoccus* sp., and *Arthrobacter* sp.) that correlated with fungal pathogen load were consistent across broad to fine spatial scales, possibly signaling their functional importance as taxa composing the skin microbial assemblage. Disease state was not incorporated into this study due to observation inconsistencies by multiple researchers, the possibility of inaccurately mistaking a lesion as SFD, and tentative development of detectable signs in a qPCR-positive snake after it was sampled and released. Accurate quantification of disease state is likely an important explanatory factor of the skin microbial assemblage and could be more accurately assessed in a controlled vivarium study.

Predictor variables of the snake skin microbiota differed between extents, illustrating the importance of incorporating scale into experimental design to predict biologically and ecologically meaningful patterns. For example, host snake species was predictive of the skin microbial assemblage across all extents, whereas geographic location was weakly predictive of microbial assemblages at a broad extent but stronger at the microscale (Fig. 1). Similar trends have been observed in bats, where the skin microbiota was significantly influenced by habitat (ecomode), host species, and between geographic regions (e.g., states [63]), demonstrating how regional environmental differences play a significant role in skin microbial assemblage composition. In addition, Loudon et al. [64] determined that salamander skin is a selective medium for specific bacterial taxa from their environment, highlighting the intricacies between environmental and species-specific microbial selection. We found that the snake skin microbiome was composed of many bacteria that exist in the surrounding environment, although structure and diversity varied and was largely host species specific. Perhaps, snake skin also serves as a selective medium for particular microbes from the environment as observed in this study, although testing for selective processes in microbial community ecology would require careful lab-based manipulation experiments. Ecomode was a predictor of skin microbial assemblages across all scales, except for the Blue Ridge Mountain ecoregion (likely due to limited sample size). To date, few studies have examined how fundamental niches among species may influence skin microbial assemblages. Price et al. [65] observed distinct bacterial community composition shifts in juvenile Green Sea Turtles (*Chelonia mydas*) when transitioning from neritic to pelagic habitats, suggesting both diet and environment may have a significant effect on microbial communities. Other studies have observed bacterial variation across plant habitat classes, e.g., leaves, stems, roots, soils (diagnosed as niches within a single organism) irrespective of genotype [66]. Among vertebrates, ecomode may be a novel predictor of observed differences in skin microbial assemblages. We found significant interactions between ecomode and snakes positive for *O*. *ophiodiicola*, indicating that the SFD pathogen may not have an equal effect among ecomodes across spatial comparisons. Understanding fungal pathogenicity by snake ecomode may help target surveillance efforts or how to better approximate required sample sizes during SFD studies in a given habitat. Future studies should examine whether shifts occur in the skin microbiota of snakes during different developmental stages, potentially elucidating periods of increased pathogen susceptibility.

SFD clearly represents a growing global conservation concern for snake species [67]. The perceived recent emergence of this fungal pathogen could be due to increased awareness of the disease, changing climatic variables, introduction of an exotic pathogen into a naive host population, or other uncharacterized factors. Nevertheless, snake species are of particular significance, as they are indicators of trophic dynamics and ecosystem health [68], and yield potential benefits to humans, such as the reduction of disease-carrying rodents [69].

This study characterized the skin bacterial assemblage of snakes and its interaction with the causative agent of SFD across the Southern United States. A more comprehensive dataset encompassing a continuous geographic range across a broader temporal period will aid in the development of models for at-risk snake populations. Predictive conservation-based models could yield targeted treatment or management techniques aimed at high-risk areas and aid in the preservation of species critical to ecosystem processes.

### Data accessibility

All raw sequence data has been submitted to GenBank SRA under the accession number PRJNA531014. All R and mothur code have been made publicly accessible in the Supplemental Materials.

## Supplementary information

Legends for supplemental materials

Supplemental R code

Supplemental mothur code

Supplemental table 1

Supplemental table 2

Supplemental table 3

Supplemental data frame
