# Supplementary material for: Variability in snake skin microbial assemblages across spatial scales and disease states
Source: ISME J. 2019 May 7;13(9):2209–22. doi: 10.1038/s41396-019-0416-x (PMC6776063; doi:10.1038/s41396-019-0416-x)
Supplement: Supplementary file 1 — Legends for supplemental materials [file 41396_2019_416_MOESM1_ESM.docx]

**Supplemental Tables**

Supplemental table 1. Alpha and beta diversity results from all tests conducted in R for macro-, meso-, and microscale comparisons.

Supplemental table 2. Complete list of taxonomic identities for OTUs in the study.

Supplemental table 3. Snake skin swab samples collected and accompanying metadata.

**Supplemental data files**

Supplemental data file 1. Complete data frame for import into R.

Supplemental mothur code.

Supplemental R code.
